# H3N2 canine influenza virus-like particle vaccine with great protection in beagle dogs

**DOI:** 10.1128/spectrum.00445-24

**Published:** 2024-06-14

**Authors:** Fei-fei Ge, Li-pin Shen, De-quan Yang, Xian-cao Yang, Xin Li, Hai-xiao Shen, Jian Wang, Shixin Huang

**Affiliations:** 1 Shanghai Municipal Center For Animal Disease Diagnosis, Shanghai Animal Disease Control Center, Shanghai, China; Wuhan Institute of Virology, Chinese Academy of Sciences, Wuhan, China

**Keywords:** distinct branch, H3N2 virus like particle, nasal and muscular immunized beagle dogs, Th1, Th2, and Th17 immune responses, attacks

## Abstract

In 2016, a distinct branch of H3N2 canine influenza virus (CIV) emerged, which has mutations related to mammalian adaptation and has replaced previously prevalent strains. This branch poses a risk of zoonotic infection. To prevent and control H3N2 CIV, an H3N2 virus-like particle (VLP) vaccine based on the insect cell baculovirus expression system has been developed in the study. The H3N2 VLP vaccine induced high titers of hemagglutination inhibition (HI) antibodies in nasal and muscular immunized beagle dogs. Meanwhile, the VLP vaccine provided effective protection against homologous virus challenge comparable to inactivated H3N2 canine influenza virus. In addition, the intranasal H3N2 VLP vaccine induced significantly higher Th1, Th2, and Th17 immune responses, respectively (p,0.05). Importantly, intramuscular injection of VLP and inactivated H3N2 virus has complete protective effects against homologous H3N2 virus attacks. Nasal immunization with H3N2 VLP can partially protect beagles from H3N2 influenza.

**IMPORTANCE:**

A new antigenically and genetically distinct canine influenza virus (CIV) H3N2 clade possessing mutations associated with mammalian adaptation emerged in 2016 and substituted previously circulating strains. This clade poses a risk for zoonotic infection. In our study, intramuscular injection of the H3N2 virus-like particle (VLP) vaccine and inactivated H3N2 CIV confer completely sterilizing protection against homologous H3N2 canine influenza virus challenge. Our results provide further support for the possibility of developing VLP vaccines that can reliably induce immunity in animal species.

## INTRODUCTION

Dogs are the dearest companion pets of human beings, but which role the dogs play in the ecology of the influenza virus is not clear. H3N2 avian influenza virus spread to dogs around 2006 and has formed a stable lineage. In the last few years, it has posed a serious threat to the health and public health safety of dogs (1). Bodewes infected beagle dogs with H3N2 canine influenza virus (CIV) of 106 TCID50 and found that the lung area of all dogs with obvious clinical symptoms was >10% (2). It is noteworthy that H3N2 CIV can be recombined with other influenza viruses. The results showed that natural co-infection of H3N2 CIV and H1N1 pdm09 virus in the same host could lead to wild-type H3N1 virus (3). Additionally, a novel H3N2 CIV carrying the polymerase acid gene of the H9N2 avian influenza virus was isolated in Korea in 2015 (4). Therefore, dogs are potential mixing vessels for influenza viruses (5). Due to the sensitivity of dogs to infection and their close physical contact with humans, the risk of human infection with new influenza virus strains increased exponentially (6). It is reported that H3N2 originated from avian influenza virus in aquatic birds. We found that since 2016, the H3N2 virus belonging to a novel branch has been prevalent in dogs in some regions of China, completely replacing the previous strain. This H3N2 branch may originate from CIV in South Korea or the United States (7).

The most useful method to prevent influenza infection is vaccination (8, 9). At present, only the inactivated influenza vaccine (IIV) is available to prevent H3N2 or H3N8 CIV infection in dogs (10). The production of inactivated influenza vaccine depends on embryonic chicken eggs, but there are many deficiencies, including endogenous virus pollution, the insufficient supply of embryonic chicken eggs during the outbreak, and the generation of biohazardous waste (11). In this paper, we present the development of a virus-like particle (VLP) for CIV H3N2, utilizing HA and NA genes from a novel H3N2 CIV. VLP exhibits high plasticity and scalability in the development of a variety of subtype influenza vaccines that are produced in various expression systems, such as insect cell baculovirus (12), plant cell (13), and mammalian cell (14). Moreover, the harmless VLP retains the structure and immune characteristics of the natural virus. Influenza VLP vaccine can induce a comprehensive immune response and provide cross protection against the challenge of homologous and heterologous influenza viruses through various routes of administration (15, 16). Thus, the VLP influenza vaccine is deemed to be one of the most promising alternatives to the traditional inactivated vaccine.

## MATERIALS AND METHODS

### Cells and viruses

Spodoptera frugiperda 9 (sf9) insect cells (Invitrogen, USA) were maintained in Sf- 900 II serum-free medium (Gibco, USA) at 27°C in shaker flasks at a speed of 100–120 rpm. The A/canine/Shanghai/0103/2019 H3N2-subtype influenza virus (GenBank accession numbers MK758007, MK758012, MK758017, MK758022, MK758027, MK758032, MK758037, and MK758042) was cultured in specific pathogen-free eggs. Formalin was used at a final concentration of 1:4,000 (vol/vol) to inactivate the virus. This study used the inactivated virus as the HAI antigen and an intramuscular vaccine. The live virus was prepared from the allantoic fluids of infected eggs for the challenge experiment in beagle dogs.

### Generation of recombinant baculovirus

To generate the VLP, the HA and NA genes from viral strain A/canine/Shanghai/0103/2019 were optimized and synthesized by GenScript Inc. (Nanjing, China). Both genes were cloned into the pFastBac Dual Expression Vector (Invitrogen, Waltham, MA, USA; catalog number 10712024). The addition of His and Flag tags to HA and NA allowed the easy detection of their expression.

### SDS-PAGE and western blot

To determine the expression of HA and NA protein, SDS-PAGE and western blot were performed as described previously (12). Briefly, the protein samples were separated by 10% Tris-Glycine gels and stained using Coomassie Brilliant Blue for SDS-PAGE analysis. The protein bands were also transferred to nitrocellulose membranes for western blot analysis.

### Indirect immunofluorescence assay

An indirect immunofluorescence assay (IFA) was carried out to detect the expression of VLP in infected sf9 insect cells. Briefly, sf9 insect cells were infected. After incubation for 48 h, the cells were fixed with pre-cooled methanol for 10 min at 4°C. Permeabilize working solution was incubated at room temperature for 20 min and washed with phosphate buffer saline (PBS) three times for 5 min each time. The fixed cells were then incubated with the antibodies, including Fluorescein isothiocyanate-conjugated goat anti-his tag IgG (ABIOCENTER, Jiangsu, China) and Cyanine 5 (CY5)-conjugated goat anti-flag tag IgG (ABIOCENTER, Jiangsu, China). The plates were then washed with PBS. Finally, the cells were stained with 4',6-diamidino-2-phenylindole (DAPI) and visualized for fluorescence under a fluorescence microscope.

### Electron microscopy

The obtained VLP suspension and H3N2 virus (A/canine/Shanghai/0103/2019) were adsorbed onto a carbon parlodion-coated copper grid for 2 min. Excess suspension was removed by blotting with filter paper, and the grid was immediately stained with 1% phosphotungstic acid for 10 min. Excess stain was removed by filter paper, and the samples were examined using a transmission electron microscope (Hitachi, Japan).

### Preparation of H3N2 VLP

After 4 days post-infection, cell culture supernatants were clarified by centrifugation (2,000 × *g* for 10 min at 4°C) and harvested as a vaccine. The functionality of HA protein incorporated into VLP was quantified by hemagglutination assay (HA assay) using 1% (vol/vol) chicken red blood cells.

### Vaccination and viral challenge

Three-week-old beagle dogs were purchased from the Experimental Animal Center (Runde Biotechnology Co., Ltd, Shanghai, China). They were maintained according to the Shanghai Animal Disease Control Center’s guidelines for the care and use of laboratory animals. H3N2 VLP was examined for immunogenicity and vaccination efficacy. Twelve specific pathogen-free beagle dogs were divided into four groups. Group 1 was immunized with 26 hemagglutination activity (HAU) of H3N2 VLPs via the i.n. route; group 2 was immunized with 26 HAU of H3N2 VLPs and an aluminum hydroxide adjuvant via the i.m. route; group 3 was immunized with an inactivated H3N2 virus containing 26 HAU titer with an aluminum hydroxide adjuvant via the i.m. route. PBS with an aluminum hydroxide adjuvant immunized group (group 4) was used as a vaccine comparison control group via the i.m. route. Beagle dogs were boosted at 14 days post-initial inoculation (dpi). At 4 weeks after the booster vaccination, under Animal Biosafety Level 2 enhanced conditions, dogs were challenged intranasally with 10^6^EID_50_ of A/canine/Shanghai/0103/2019. We observed the mortality and clinical signs daily for 5 dpi and determined viral shedding patterns by EID_50_. At 5 dpi, necropsies and histology were performed on the dogs for pathological examination. All animal procedures performed in this study were reviewed, approved, and supervised.

### Hemagglutination inhibition assay

Approximately 500 µL of serum was collected from each dog and stored at −20°C until use. First, one volume of serum was mixed with three volumes of receptor-destroying enzyme (Denka Seiken Co., LTD.) and incubated for 18 h at 37°C followed by 30 min at 56°C. The antiserum titer was then determined by the hemagglutination inhibition (HI) assay.

### Cytokine assays using quantitative real-time PCR

Total mRNA was extracted using total RNA extraction kits (Magen, Guangdong, China). Sequences of primers used for quantitative real-time PCR are shown in Table 1. The results were expressed in fold change.

**TABLE 1:** Sequences of primers used for quantitative real-time PCR

Gene	Primer sequences (5′ −3′)	Accession no.
IFN-γ	F: GGGAACATGTCTGCATGATGAG	XM025476664.3
	R: GACACAAGTCATATCACCTGACACATT	
	FAM-ACTGCTCTAGAGGCATGTCAGACGCCA-TAMERA	
IL-4	F: GTCCACGGACATAACTTCAATATTACTATT	AF187322.1
	R: CTTGACAGTCAGCTCCATGCA	
	FAM-TGTTGAACATCCTCACAGCGAGAAACGAC-TAMERA	
IL-17	F: CACTTGGGCTGTGTCAATAATGA	XM055329756.1
	R: CTTCGCAGAACCAGGATCTCTT	
	FAM-TAAACTACCACATGAACTCCGTCCCCATCC-TAMERA	

### Statistical analysis

All statistical analyses were conducted using GraphPad Prism 8.0 software (GraphPad Software, Inc., La Jolla, CA, USA).

## RESULTS

### Production and characterization of H3N2 VLP

**Fig 1:**
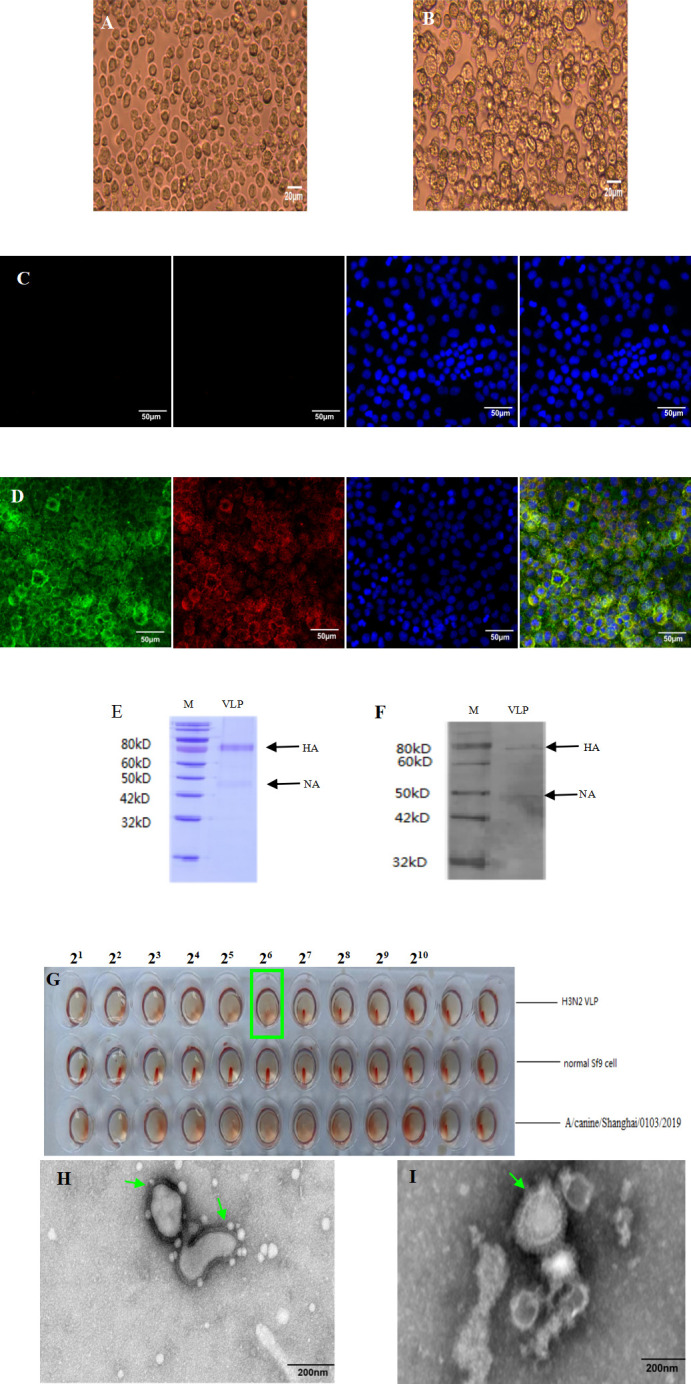
Production and characterization of H3N2 VLP. (**A**) Normal Sf9 cells. (**B**) Indicate the cytopathic effect of Sf9 cells after infection with recombinant baculoviruses rBV. (**C**) Normal Sf9 cells. (**D**) Detection of VLP in Sf9 cells by an immunofluorescence assay at 48 h post-infection. The cells were fixed and incubated with anti-his tag IgG, anti-flag tag IgG, and DAPI, respectively. (**E and F**) The expression of the HA and NA proteins on the VLP was analyzed using SDS-PAGE gels with coomassie blue staining and validated by western blot using mouse serum antibody against H3N2 CIV (A/canine/Shanghai/0103/2019). (**G**) The hemagglutination activity of H3N2 VLPs was assessed with a hemagglutination assay. The hemagglutination activity of the H3N2 VLP was 2^6^ (green square). (**H and I**) Negative staining electron microscopy of H3N2 CIV (A/canine/Shanghai/0103/2019) (green arrows) and the H3N2 VLP (green arrows).

Recombinant bacmid was transfected into Sf9 cells to obtain recombinant baculovirus expressing HA and NA genes. Compared with the uninfected sf9 cells, sf9 cells were clustered and fragmented after infection. No cytopathy occurred in the control well (Fig. 1A and B). The specific fluorescence of VLP was observed in the infected sf9 cells by the IFA method but not in the control cells (Fig. 1C and D). The generation of VLP was confirmed by SDS-PAGE and western blotting (Fig. 1E and F). The molecular weights of HA and NA proteins were 70 kDa and 46 kDa, respectively. The hemagglutination activity of H3N2 VLP reached 26 (Fig. 1G). The size and morphology of VLP and virus were observed by means of a transmission electron microscope (Fig. 1H and I). The average size of VLP is 100 nm. The morphology of VLP was similar to that of influenza virus particles, and spikes were observed on the spherical surface, simulating the HA protein of influenza virus on natural virus particles. The results exhibited that H3N2 VLP has been successfully assembled, and its shape and size were similar to those of natural influenza virus particles.

### Immune responses to vaccination with CIV H3N2 VLPs

**Fig 2:**
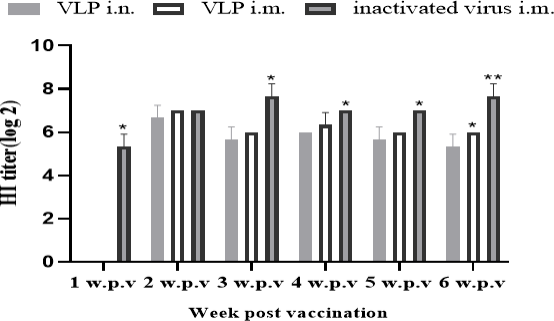
Mean serum HI titers induced in beagle dogs after the first immunization. HI titers against the homologous antigen were determined 1, 2, 3, 4, 5, and 6 weeks post vaccination. Results were expressed as the mean ± SD (*n* = 3; Note: data marked * differentiated significantly, *P < 0.05*).

To compare the immunogenicity of the VLP vaccine and inactivated virus, we determined serological immune responses 1, 2, 3, 4, 5, and 6 weeks after the first vaccination. There was no detectable antibody prior to vaccination in all groups. As shown in Fig. 2, these results suggest that the three immunized groups can induce HI titer of 2^5^–2^8^ at 2, 3, 4, 5, and 6 weeks after the first vaccination (Fig. 2).

### Protective efficacy of CIV H3N2 VLPs

**Fig 3:**
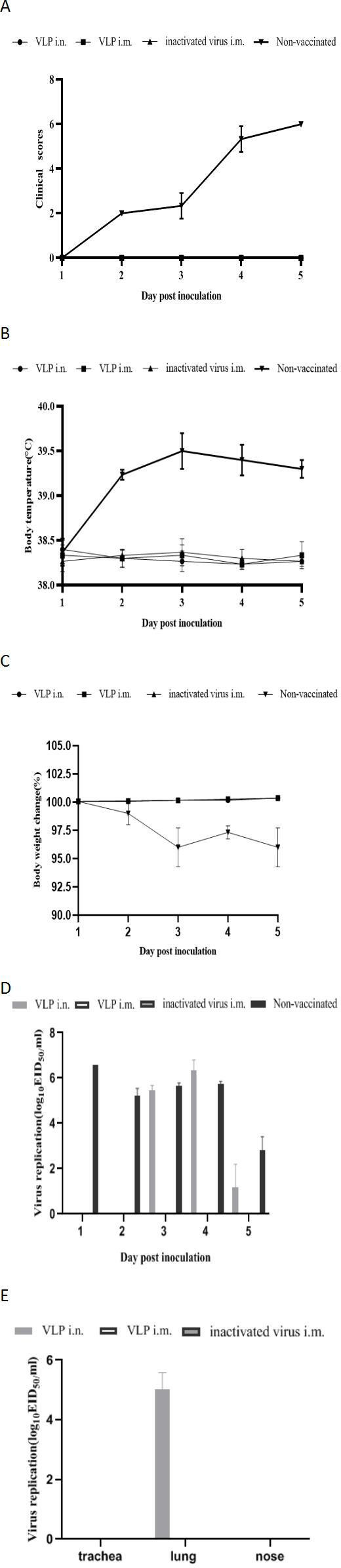
Mean clinical scores were recorded in beagle dogs on days 1–5 post-challenge. (A) Mean body temperature was recorded in beagle dogs on days 1–5 post-challenge. (B) Mean body weight change was recorded in beagle dogs on days 1–5 post-challenge. (C) Quantification of the virus in the nasal swab samples was determined by EID_50_. (D) Quantification of the virus in the trachea, lung, and nose was determined by EID_50_. (E) Results were expressed as the mean ± SD (*n* = 3).

**Fig 4:**
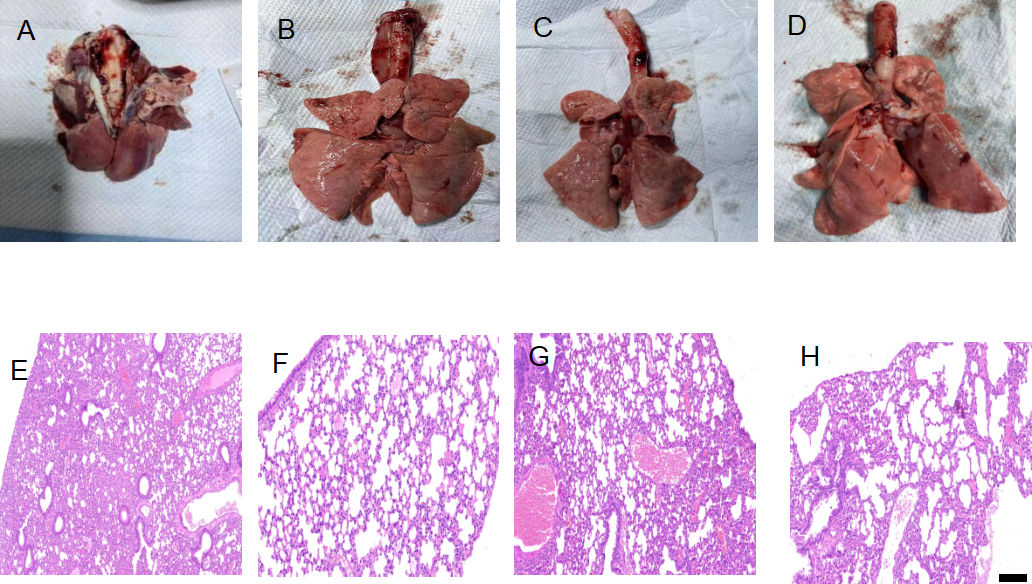
Gross and histopathological changes in the lungs infected with CIV. Gross lesion and hematoxylin-and-eosin-stained lung sections (scale bar = 200 µm) from dogs at 5 days post-infection are shown. (**A and E**) Unvaccinated dog. (**B and F**) Intranasal VLP. (**C and G**) Intramuscular VLP. (**D and H**) Inactivated H3N2 influenza virus.

In the vaccinated groups, no clinical signs of infection were observed during the observation period following intranasal challenge with CIV H3N2 among the three immunized groups. However, in the non-vaccinated group, all infected dogs exhibited clinical signs of respiratory disease, including nasal discharge, ocular discharge, coughing, anorexia, and depression. Clinical signs were evident as early as 2 dpi. Nasal discharge and coughing were the predominant clinical signs. Anorexia and depression began at 2 dpi and persisted until 5 dpi. Clinical symptoms were observed on days 1–5 post-challenge (Fig. 3A). At 2–5 dpi, infected dogs developed a transient clinical fever (≥39.1°C). The mean body temperature of the vaccinated dogs was within the normal range (Fig. 3B). The percent of weight change was based on the starting weight of 0 dpi. Body weight loss was not noted in the vaccinated groups, but slight body weight loss was noted in the unvaccinated group (Fig. 3C). The non-vaccinated group showed the highest virus titers in the nasal swabs. Notably, at 1–5 dpi, the two intramuscular vaccinated groups showed no viral excretion. The intranasal vaccinated group showed virus excretion at 2–4 dpi (Fig. 3D). As shown in Fig. 3E, virus titers were evaluated from turbinates, tracheas, and lungs of infected dogs at 5 dpi. No virus was detected in all three tissues of the two intramuscular vaccinated groups. The virus was only detected in the lung of the intranasal vaccinated group. Gross necropsy revealed diffuse dark red consolidated areas in the lung lobes of non-vaccinated dogs (Fig. 4A). Although petechial hemorrhagic lesions were observed in the lungs of dogs vaccinated with intramuscular VLPs, inactivated H3N2 subtype influenza virus, and intranasal H3N2 VLP, dogs vaccinated did not exhibit significant morphological changes in the lungs (Fig. 4B through D). Unvaccinated dogs showed severe interstitial pneumonia, and all vaccinated dogs showed slight histopathological changes (Fig. 4E through H).

### H3N2 VLP enhances Th1-type, Th2-type, and Th17-type immune responses

**Fig 5:**
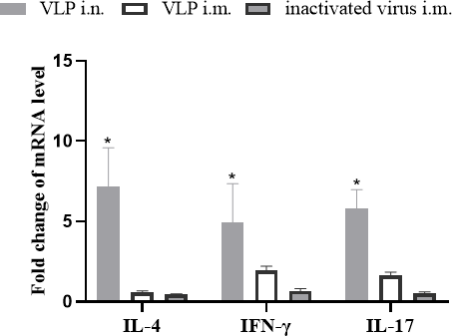
Cytokine expression levels in PBMCs of beagle dogs (*n* = 3). The mRNA expression levels of IFN-γ, IL-4, and IL-17 after virus stimulation (Note: data marked * differentiated significantly, *P < 0.05*).

To evaluate the effect of VLP on the immune response types, the levels of the cytokines IFN-γ, IL-4, and IL-17, associated with Th1-type, Th2-type, and Th17-type immune responses, respectively, were determined. Beagle dogs were immunized with H3N2 VLP by intranasal and intramuscular injection, and peripheral blood mononuclear cells (PBMCs) were isolated at 19 days after immunization and stimulated with H3N2 CIV *in vitro*. For stimulation, the results indicated that dogs receiving intranasal H3N2 VLP vaccine had higher IFN-γ, IL-4, and IL-17 mRNA expression levels than the other two immunized groups (Fig. 5). Overall, these results indicate that intranasal H3N2 VLP augments Th1-type, Th2-type, and Th17-type immune responses compared to intramuscular immunization.

## DISCUSSION

H3N2 CIV is an avian influenza virus that originated from aquatic birds. We found that since 2016, a new branch of the H3N2 virus has been prevalent in dogs in some regions of China, completely replacing the previous strain. The H3N2 branch can be derived from CIV in South Korea or the United States (17). However, the sparse sequence data of isolates from South Korea and the United States, as well as the lack of CIV H3N2 sequence in these countries after 2016, hindered the ancestral identification of this branch. Unlike the geographic clustering of H3N2 CIV strains observed during transmission in the United States (18), H3N2 CIV strains isolated in Beijing (North China), Shanghai and Nanjing (southeast), and Xi'an (West) from 2016 to 2017 have high genetic homology.

Canine influenza is a canine infectious respiratory disease caused by two subtypes of CIV (H3N2 and H3N8). At present, only IIV can be used to prevent CIV. History has proved that live attenuated influenza vaccine has better immunogenicity and protective effect than IIV. Here, we constructed the CIV H3N2-VLP using the HA and NA genes of the circulating CIV H3N2 strain. Our results showed that H3N2 CIV VLP had similar protective effects on beagles compared with inactivated H3N2 influenza virus.

Currently, only IIVs are available commercially to prevent infection of either H3N2 or H3N8 CIVs in dogs (10). Vaccination of dogs with CIV H3N2 VLP and IIV uses two i.m. injections with 1 mL of vaccine dose in our study. CIV H3N2 VLP by intramuscular immunization confers complete protection against virus infection compared with CIV H3N2 IIVs. Intranasal immunization is a desirable delivery method for providing optimal immunity to influenza A virus because it leads to the generation of mucosal immune responses, creating an immune barrier at the site of potential infection (19), as well as systemic humoral responses, cellular immunity (20–24). However, intranasal CIV H3N2 VLP did not induce complete protection against CIV H3N2 challenge at least at the times post-infection we have evaluated. Therefore, the intranasal adjuvant and dose will be investigated in further studies.

There are advantages to a VLP vaccine approach. VLPs mimic virus particles, presenting multiple antigenic epitopes that stimulate a diverse set of immune responses. In addition, the use of VLP vaccines could help address the safety concerns associated with live-attenuated and inactivated whole-virus vaccines (25, 26). In the present study, we describe the development of an H3N2 influenza VLP vaccine composed of only two influenza virus structural proteins, HA and NA, which were derived from CIV H3N2. VLP vaccine elicited great antibodies to the virus, as shown by HI activity, and protected against wild-type virus infection. Our results provide further support for the possibility of developing VLP vaccines that can reliably induce immunity in animal species.
